# Linking Native and Invader Traits Explains Native Spider Population Responses to Plant Invasion

**DOI:** 10.1371/journal.pone.0153661

**Published:** 2016-04-15

**Authors:** Jennifer N. Smith, Douglas J. Emlen, Dean E. Pearson

**Affiliations:** 1 Division of Biological Sciences, University of Montana, Missoula, Montana, United States of America; 2 Rocky Mountain Research Station, United States Department of Agriculture, Forest Service, Missoula, Montana, United States of America; Scientific Research Centre, Slovenian Academy of Sciences and Arts, SLOVENIA

## Abstract

Theoretically, the functional traits of native species should determine how natives respond to invader-driven changes. To explore this idea, we simulated a large-scale plant invasion using dead spotted knapweed (*Centaurea stoebe*) stems to determine if native spiders’ web-building behaviors could explain differences in spider population responses to structural changes arising from *C*. *stoebe* invasion. After two years, irregular web-spiders were >30 times more abundant and orb weavers were >23 times more abundant on simulated invasion plots compared to controls. Additionally, irregular web-spiders on simulated invasion plots built webs that were 4.4 times larger and 5.0 times more likely to capture prey, leading to >2-fold increases in recruitment. Orb-weavers showed no differences in web size or prey captures between treatments. Web-spider responses to simulated invasion mimicked patterns following natural invasions, confirming that *C*. *stoebe*’s architecture is likely the primary attribute driving native spider responses to these invasions. Differences in spider responses were attributable to differences in web construction behaviors relative to historic web substrate constraints. Orb-weavers in this system constructed webs between multiple plants, so they were limited by the overall quantity of native substrates but not by the architecture of individual native plant species. Irregular web-spiders built their webs within individual plants and were greatly constrained by the diminutive architecture of native plant substrates, so they were limited both by quantity and quality of native substrates. Evaluating native species traits in the context of invader-driven change can explain invasion outcomes and help to identify factors limiting native populations.

## Introduction

The introduction of exotic organisms can result in immediate and substantial reorganization of native species within recipient communities [1]. Yet, the mechanisms driving native species responses to invasions are not well understood. Advances have been made in elucidating how invaders negatively impact native species via competition, consumer interactions, and natural enemies [2–4]. However, some exotic species generate a range of positive responses in native species [5, 6]. The positive effects of invaders on resident species has received far less attention, yet positive interactions are critical aspects of community structuring (e.g., [7]). Understanding how anthropogenic perturbations like biological invasions will affect native communities requires accounting for the full range of potential responses of native species from negative to neutral to positive [8]. Moreover, understanding the mechanisms that underlie native species responses to invasions can help elucidate those factors structuring native communities.

Niche theory proposes that a species’ presence or absence and relative abundance within a community is determined by how its functional traits interact with biotic and abiotic processes to determine its fundamental and realized niches [9–11]. This body of theory should also be applicable for understanding native species responses to invasions and other anthropogenic perturbations. However, an important caveat in applying niche theory to invasions is that invasions more often involve “community reassembly” in response to a new community member, rather than complete community assembly as is often idealized in community theory. That is to say, the initial composition and relative abundance of native system has already been determined by regional and local filters, so that the introduction of an exotic organism creates a biotic perturbation that reorganizes native species within an already defined parameter space. Within this framework, it should be possible to understand how native species respond to an invasion by evaluating how the invader’s traits alter the system (biotically and/or abiotically) and how native species traits align or fail to align with these new conditions. While functional traits of invaders have been examined extensively in an effort to predict invader success [12], few studies have explored how functional traits of native species might help predict their responses to invasion in the context of fitting those traits to invader-driven change [8].

Native web spiders represent an important guild of predators that are strongly influenced by exotic plant invasions [13–16], an outcome that can have profound food web ramifications [17]. These spiders exhibit a range of web-building strategies that represent extended phenotypes or functional traits which are tightly linked to their ecological roles in native communities [18, 19]. Because different web designs require specific substrate attributes, web spiders are sensitive to habitat modification [16, 18, 20]. Hence, web spider responses to plant invasions should be predictable, at least in part, as a function of how plant invasions alter web substrates in relation to specific web-building strategies.

In the intermountain grasslands of the western United States, invasions by the perennial forb spotted knapweed (*Centaura stoebe* L. formerly *C*. *maculosa*) have been linked to a dramatic reshuffling of native web spider communities ([13], Smith *pers*. *obs*.). In invaded areas, native web spiders primarily use *C*. *stoebe*’s persistent, standing dead stems as web substrate, secondarily moving to new growth later in the summer as the plants get taller and intermingle with previous years’ dead stems [13]. Late stage *C*. *stoebe* invasions may result in an approximate 20-fold increase in orb weaver densities and a near 80-fold increase in irregular web spider densities [13]. These changes in native spider abundance have been attributed to a shift in plant architecture associated with the fact that *C*. *stoebe* and other invading forbs generate taller, more expansive, and far more abundant flowering stems than the native forbs commonly used by native spiders [13, 21]. However, studies to date have been observational, comparing spider populations between invaded and uninvaded grasslands. Because *C*. *stoebe* invasions greatly alter native plant, vertebrate, and invertebrate communities [22–24], all of which could affect native spider populations [14–16], the specific mechanism by which *C*. *stoebe* invasion affects spider populations is not certain. As this example demonstrates, only so much can be learned from biological invasions using observational approaches. Yet, there have been remarkably few large-scale *in situ* invasion experiments demonstrating the causal mechanisms underlying invasion outcomes.

Here, we set out to disentangle the effects of *C*. *stoebe* invasion on native spider species in this system by conducting a large-scale simulated invasion. In particular, we wished to determine whether linking novel aspects of the invader’s traits with native species traits could explain the natives’ responses to invasion. Our objectives were to (1) experimentally determine whether the invader’s architecture caused native web spider population changes observed in natural invasions and (2) evaluate whether differences in web construction strategies could explain the differences in population responses of native spider species to *C*. *stoebe* invasion. To accomplish this, we simulated large-scale *in situ* invasions by introducing only dead stems of *C*. *stoebe* into native grasslands in order to isolate the plant’s architecture from other effects of invasion. We compared web construction, prey capture rates, reproduction, and population densities of spiders on these simulated invasion plots with adjacent control plots (no stems added), with initial spider densities on all plots standardized by removing native spiders and seeding plots with known spider densities. We predicted that spider population responses to the simulated invasion treatments would mimic those observed in natural invasions if invader architecture was the primary factor driving spider responses. We also predicted that population and demographic responses of different spider species to the treatments should link to differences in web construction strategies if web construction was the key trait determining native species responses to invasion.

## Materials and Methods

### Ethics statement

Fieldwork was conducted with permission on public lands managed by Montana Fish, Wildlife, and Parks; Montana Department of Natural Resources and Conservation; U.S. Fish and Wildlife Service; the Bandy Ranch deeded to the Montana Forest and Conservation Experiment Station at the University of Montana; and private lands owned by Verne Imboden. All methods used in this study complied with the requirements of the Institutional Animal Care and Use Committee of the University of Montana, which does not require protocol review or permits for research with invertebrates. During the experiment we avoided any unnecessary harm, suffering, or distress to study subjects. This research did not involve endangered or protected species.

### Study system

**Fig 1:**
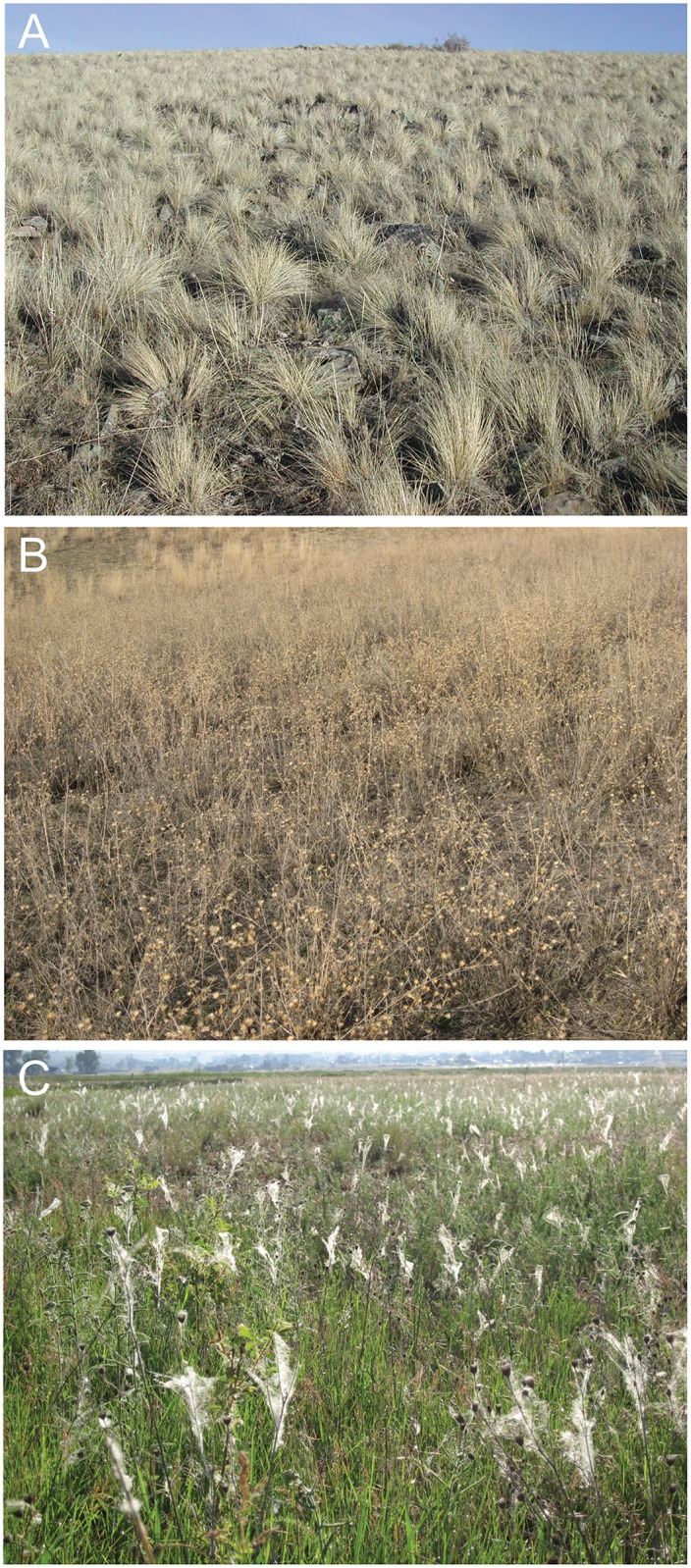
Plant architecture changes following *C*. *stoebe* invasion in Montana. (A) Native, uninvaded grasslands dominated by bunchgrasses; (B) grasslands invaded by *Centaurea stoebe*; and (C) native spiders building webs on *C*. *stoebe* plant architecture.

We conducted our research in the semi-arid, low-elevation grasslands of the Rocky Mountains in the Blackfoot Valley of western Montana, USA. These grasslands are dominated by one native bunchgrass (*Festuca capestrus* Rydb., formerly *F*. *scabrella*; Fig 1A), with native forbs comprising much of the plant diversity. Forbs, primarily their standing dead stems, serve as the dominant web substrates for native spiders in this habitat. However, native forbs are highly ephemeral, flowering in the wetter months of May and June and senescing by mid-July, leaving few residual standing stems for most of the year. They also generate flowering stems that are shorter, less expansive, and less abundant than *C*. *stoebe* (and other invaders), thereby providing lower quality and less plentiful substrates for web-building spiders ([13, 21], Fig 1B and 1C). Web-building spider communities in this system are fairly simple, being comprised of only a few species of cribellate or irregular web spiders (Family Dictynidae: *Dictyna major* Menge and *D*. *coloradensis* Chamberlin), orb weaving spiders (Family Araneidae: *Aculepeira packardi* Thorell; Family Tetragnathidae: *Tetragnatha laboriosa* Hentz), and funnel web weavers (Family Agelenidae), none of which are abundant [25]. Of these groups, *Dictyna* spp., *A*. *packardi*, and *T*. *laboriosa* were the most abundant in our study areas.

*D*. *major* and *D*. *coloradensis* dominate most web-building spider communities in western Montana grasslands [13]. These two species are ecologically similar [26] and indistinguishable in the field (J. Slowik, University of Alaska Fairbanks, *pers*. *comm*.), thus we treat them as a species complex and refer to them as *Dictyna*. However, identification of specimens from our populations indicate about 95% of the spiders are *D*. *coloradensis*. *Dictyna* are small spiders (female mean total body length approximately 3 mm for *D*. *major* and 3.8 mm for *D*. *coloradensis*) that overwinter in the plant litter as sub-adults, emerging in April and May as the temperature warms [27, 28]. These spiders breed and begin to produce egg sacs (1–5) by the end of June through mid-July. Spiderlings emerge and disperse by ballooning in mid- to late-July. *Dictyna* prey mostly on small insects (e.g. Hymenoptera, Diptera), which they capture and retain in their webs [27].

The orb-weavers are the second most abundant group of web-building spiders in these grasslands, with *Aculepeira packardi* being the most abundant. These are larger spiders (female mean total body length 10.77 ± 2.19 mm) [29]. Sub-adults overwinter in plant litter, emerging in spring. *A*. *packardi* construct large, orb webs by attaching their silk to multiple plants, suspending their web between plant substrates. Adults become sexually mature in early August, with females producing 1–3 egg sacs by late August into September [30]. Spiderlings emerge and immediately disperse from their mother’s web via ballooning by mid-September. *Tetragnatha laboriosa* is the second most common orb-weaver present, albeit in much lower abundances. This species has a long, slender body (female mean total body length 6.17 ± 0.43 mm) and long legs [29]. *T*. *laboriosa* construct nearly horizontal orb-webs and in this system suspend their webs between multiple plant substrates [31]. Reproductive timing is similar to *A*. *packardi* [31]. Both orb-weavers have a broad spectrum of prey items (i.e. Orthoperta, Homoptera, Diptera, Coleoptera, and Hymenoptera), which can range in size by nearly two orders of magnitude [29, 31, 32].

### Experimental design and sampling methods

**Fig 2:**
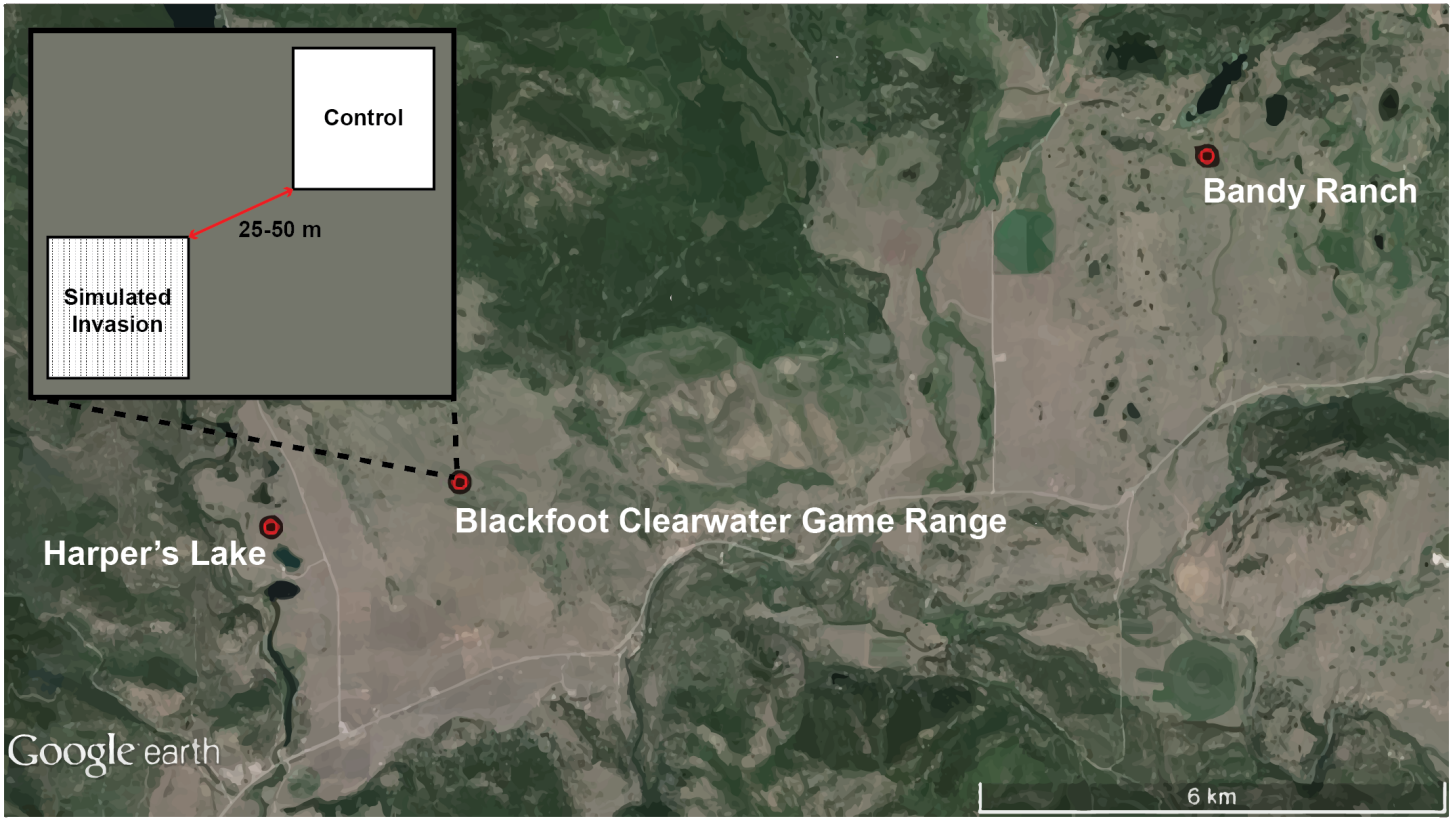
Map showing locations of the three study sites (Harper’s Lake, Blackfoot Clearwater Game Range, Bandy Ranch) located in the Blackfoot Valley of western Montana, USA. The spatial layout of simulated invasion treatment and control plots is also shown. Dashed lines on treatment plot represent survey transects and locations of introduced *C*. *stoebe* stems. Distances between treatment and control plots ranged between 25–50 m at all sites. Map Data: Google, Landsat.

We simulated invasions by introducing dead *C*. *stoebe* stems into native, uninvaded, grasslands at three sites in May 2011 (Fig 2). At each site three 0.25 ha (50 x 50 m) paired plots were established; one plot received the simulated invasion treatment of 1250 dead *C*. *stoebe* stems with seed heads removed (to prevent invasion), the other plot served as a control with no stems introduced (Fig 2; N_simulated invaion_ = 3, N_control_ = 3). *C*. *stoebe* stems were collected locally and set out in a grid of 25 rows spaced 2 m apart, with 100 stems placed 0.5 m apart in each row. Stems were replaced as needed throughout the study to maintain 2500 stems on simulated invasion plots. Due to logistical constraints of conducting a large-scale *in situ* experiment, stem densities simulate a light or early invasion compared with heavily invaded areas where stem densities can average 320,000 stems/0.25 ha [21].

Two weeks after knapweed stems were erected on the simulated invasion plots (mid-May 2011), spider densities were standardized across treatment and control plots by removing all web-building spiders and reseeding plots with known numbers of spiders. This is feasible because spider densities in these grasslands are extremely low and webs are very visible on vegetation during early morning hours (i.e. low light, dew covered webs). Removal and reseeding of spiders was completed at both control and treatment plots at a given site on the same day, with all sites completed over 3 consecutive days. Additionally, equal search effort was given to treatment and control plots, thus if spiders happened to be missed the likelihood should be equivalent between paired plots. Each plot was then seeded with 20 female *Dictyna* and 10 female *A*. *packardi* (= initial seeding densities). Spiders were placed on native vegetation throughout each plot. These species were chosen as focal subjects because they represent the two distinct web-building groups that show different response to *C*. *stoebe* invasion. Differences in seeding densities reflect relative abundances of these spiders in the native grasslands of western Montana (J. Smith, pers. obs.). *T*. *laboriosa* was not experimentally added, but naturally colonized and so was quantified where possible.

Sites were sampled during summers 2011 and 2012 with three sampling events taking place each year (June, July, and August) to determine demographic and population responses of each species and to identify potential mechanisms underlying community-level responses to invasion. During each sampling period, abundance of each species was censused by walking 50 transects per plot (spaced 2 m apart), finding occupied webs, counting the number of spiders in each web, and noting the sex (male, female) and maturity (adult, juvenile) of observed individual(s). The length and width of each observed web was measured to the nearest 0.5 cm with a tape measure following Pearson 2009 [13]. These data were then used to calculate web area based on the geometry of a triangle [26]. In each web we counted whole prey items and each individual prey item was measured to the nearest 0.005 mm with digital calipers. The number of spiderlings present in each web was also recorded. Web area, prey number and size, and spiderling number were assessed during all sampling periods in 2011. These metrics were only observed during June 2012 in the second year of study due to increased spider abundances making collection of these metrics time prohibitive in July and August. The abundance of available prey on treatment and control plots was assessed in mid-July 2012 by sweep net sampling. Ten equally spaced 50 m transect sweep net samples were collected on each plot by sweeping once every meter (= 50 sweeps) at 0.5 m above the ground [33]. Sweep net sampling took place between rows of stems on simulated invasion plots. Abundance of available prey in each sweep sample was tallied by counting only those invertebrate species which were previously observed in webs during surveys.

### Analyses

The change in abundance of focal spider species (*Dictyna*, *Aculepeira*, *Tetragnatha*) from initial seeding densities to final sampling densities (August 2012 abundance—seeding densities) in response to simulated invasion treatment (simulated invasion vs. control) were analyzed using a permutational multivariate analysis of variance (PERMANOVA) in the R statistical package version 3.2.2, with Euclidean distance and 720 permutations using the Adonis function (package vegan) [34–36]. This analysis allowed us to assess population-level effects of the simulated invasion treatment by taking into account the potential interdependence of the species’ abundance responses while also enabling simultaneous investigation of species-specific population responses. It was selected because it is robust to heterogeneity of dispersion between groups, small sample sizes, and negative data [35]. Demographic effects (i.e. web size, prey captures, overwinter survival, and reproduction) of the simulated invasion treatment were analyzed on a species-specific basis in *Dictyna* and *Aculepeira* using linear mixed effects models (LMM), which allowed us to account for differences between sites. The effect of invasion treatment on average web area was analyzed with LMMs using PROC GLIMMIX in SAS version 9.2, with treatment and year as fixed effects, and site as a random blocking factor, using a log normal distribution to meet model assumptions [37]. We tested for differences in average web area across sampling periods (June, July, and August) in 2011 using LMMs (package nlme) in R with plot and sampling period as fixed effects and site as a random blocking factor, using a log normal distribution [36, 38]. The effect of simulated invasion on number of prey captured per web was analyzed with LMMs using PROC GLIMMIX in SAS, with treatment as a fixed effect and site as a random blocking factor, using a negative binomial distribution. The effect of simulated invasion on the probabilities of capturing prey and capturing large prey (≥ 3 mm for *Dictyna*, ≥ 4 mm for *Aculepeira*) was done with LMMs using PROC GLIMMIX in SAS, with treatment as a fixed effect and site as a random blocking factor using logistic regression with a binomial distribution [37]. Available prey was analyzed using LMM in R (package nlme) with treatment as a fixed effect and site as a random blocking factor [36, 38]. Analysis of overwintering survival or the change in abundance between year one and two of study (June 2012 –August 2011) was only conducted for *Dictyna* due to logistical issues that prevented sampling *Aculepeira* populations in 2011 after recruitment. This was done using a LMM in R (package nlme) with treatment as a fixed effect and site as a random blocking factor [36, 38]. Analysis of the number of spiderlings per female was only conducted for *Dictyna* in 2011 due to sampling constraints explained further in Results. This was done with a LMM using PROC GLIMMIX in SAS, with treatment as a fixed effect and site and sampling period as random blocking factors using a negative binomial distribution [37]. Mean number of juveniles per female $\left(=\frac{\#\text{ juveniles August }2012}{\#\text{ reproducing females June }2012}\right)$ was analyzed using LMMs in R (package nlme) with treatment as a fixed effect and site as a random blocking factor [36, 38]. For all analyses, we inspected plots of residuals against predicted values to assess model fit and found no indications of significant deviations from assumptions of linearity and homoscedasticity.

## Results

**Fig 3:**
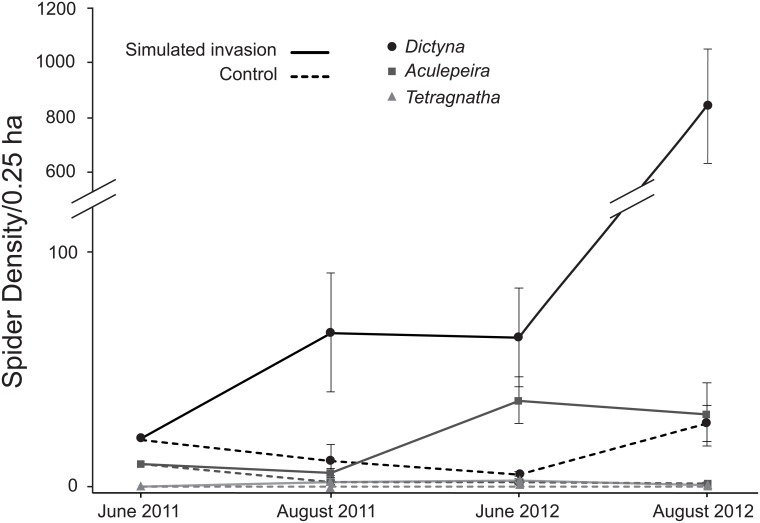
Spider density (mean ± SE) on plots where *C*. *stoebe* dead stems were introduced (simulated invasion) compared to control plots for three dominant grassland spiders over two-years (2011–2012). Spider density on treatment plots was >30x higher in *Dictyna* and >23x higher in *Aculepeira* compared to controls (see Results).

Web-building spider populations were 31 times higher on simulated invasion plots (N = 3, $\bar{x}\;=872.333\pm \;208.132\text{ SE}$) compared to controls (N = 3, $\bar{x}\;=28.667\pm \;7.265\text{ SE}$) at the end of the study (PERMANOVA: *F*_1,4_ = 13.72, *P* = 0.1; Fig 3; S1 Table). *Dictyna* populations increased dramatically in response to the treatment with final densities 31 times higher on simulated invasion plots ($\bar{x}=840.667\pm \;212.289\text{ SE}$) compared to controls ($\bar{x}=27.333\pm \;7.881\text{ SE}$, *F*_1,4_ = 13.7, *P* = 0.098). Similarly, final *Aculepeira* densities were 23 times higher on simulated invasion plots ($\bar{x}=31.0\pm \;13.429\text{ SE}$) compared to controls ($\bar{x}=1.333\pm \;0.667$, *F*_1,4_ = 4.87, *P* = 0.113). *Tetragnatha* only colonized simulated invasion plots.

**Fig 4:**
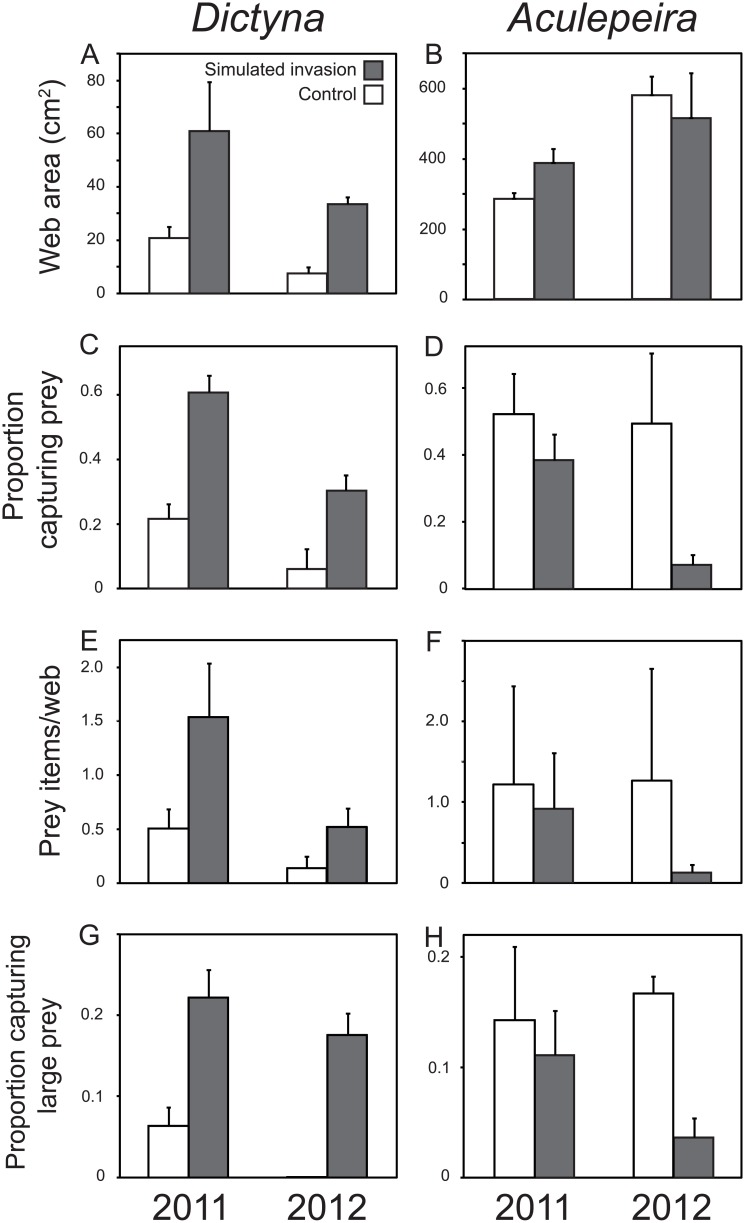
Responses of *Dictyna* (left) and *Aculepeira* (right) spiders to simulated *C*. *stoebe* invasion in terms of web area (mean ± SE, raw data; A, B), proportion of females capturing prey (mean ± SE; C, D); number of prey captured per web (mean ± SE; E, F), and the proportion capturing large prey (mean ± SE; G, H). *Dictyna* on simulated invasion plots constructed larger webs that increased their likelihood of capturing prey compared to webs on control plots. We observed no differences in any of these investigated metrics in *Aculepeira* between simulated invasion and control plots, however *Aculepeira* on control plots tended to be more likely to capture prey (see Results).

Species-specific demographic responses to the simulated invasion treatment appeared to be related to web-building strategy. *Dictyna* constructed larger webs on treatment versus control plots (Table 1, Fig 4A). Although *Dictyna* webs were larger in 2012 compared to 2011, the pattern of larger webs on simulated invasion plots held in both years (Table 1). *Aculepeira* web size did not differ by invasion treatment, year, or their interaction (Table 1, Fig 4B). Web size data was pooled in 2011 as webs of both spider species do not persist across sampling periods (~1 month) and web area did not differ between sampling periods (*Dictyna $F_{1,153}^{period}=1.835$*, *P* = 0.178; *Aculepeira $F_{1,28}^{period}=0.907$*, *P* = 0.349).

**Table 1:** Results from linear mixed effects models testing the effects of simulated invasion treatment and year on web area, proportion capturing prey, number of prey per web, proportion capturing large prey, number of spiderlings per female, and number of juveniles per female.

		Treatment effect			Year effect			Treatment x Year		
Species	Spider species responses	df	*F*	*P*	df	*F*	*P*	df	*F*	*P*
*Dictyna*	Web area	1, 4	57.23	0.002	1, 4	21.40	0.010	1, 4	0.34	0.591
	Proportion capturing prey	1, 4	9.73	0.036	1, 4	6.17	0.068	1, 4	0.03	0.877
	# Prey/web	1, 4	4.51	0.101	1, 4	10.07	0.034	1, 4	0.07	0.811
	Proportion capturing large prey	1, 4	0.00	0.969	1, 4	0.00	0.974	1, 4	0.00	0.975
	# Spiderlings/female	1, 4	4.27	0.108						
	# Juveniles/female	1, 2	62.96	0.016						
*Aculepeira*	Web area	1, 4	0.39	0.579	1, 4	0.28	0.632	1, 4	1.76	0.276
	Proportion capturing prey	1, 4	7.75	0.069	1, 4	3.88	0.143	1, 4	3.12	0.176
	# Prey/web	1, 4	1.82	0.270	1, 4	1.25	0.345	1, 4	1.35	0.329
	Proportion capturing large prey	1, 4	2.02	0.250	1, 4	0.55	0.512	1, 4	1.01	0.389
	# Spiderlings/female									
	# Juveniles/female	1, 2	1.64	0.329						

The larger *Dictyna* webs constructed on treatment plots were more likely to capture prey (Table 1, Fig 4C). The likelihood of capturing prey tended to be higher in 2011 compared to 2012, but the pattern of *Dictyna* being more likely to capture prey on simulated invasion plots held in both years (Table 1, Fig 4C). Mean number of prey captured in *Dictyna* webs trended towards being greater on simulated invasion plots compared to control plots, and although *Dictyna* captured more prey in 2011 compared to 2012, there was no interaction between treatment and year (Table 1, Fig 4E). The likelihood of *Aculepeira* capturing prey was not different between control and treatment plots (though there was a trend toward higher captures on the control plots), between years, or treatment by year (Table 1, Fig 4D). There was no difference in the mean number of prey captured per web by treatment, year, or their interaction (Table 1, Fig 4F) for *Aculepeira*. *Dictyna* on treatment plots were not more likely to capture large prey (≥ 6 mm), there were no differences between years, nor was there an interaction between treatment and year (Table 1, Fig 4G). Similarly for *Aculepeira* there were no differences in the proportion of individuals capturing large prey by treatment, year, or their interaction (Table 1, Fig 4H). Abundance of available prey surveyed in July 2012 did not differ between control and treatment plots (*F*_*1*,*2*_ = 4.96, *P* = 0.156; S2 Table).

**Fig 5:**
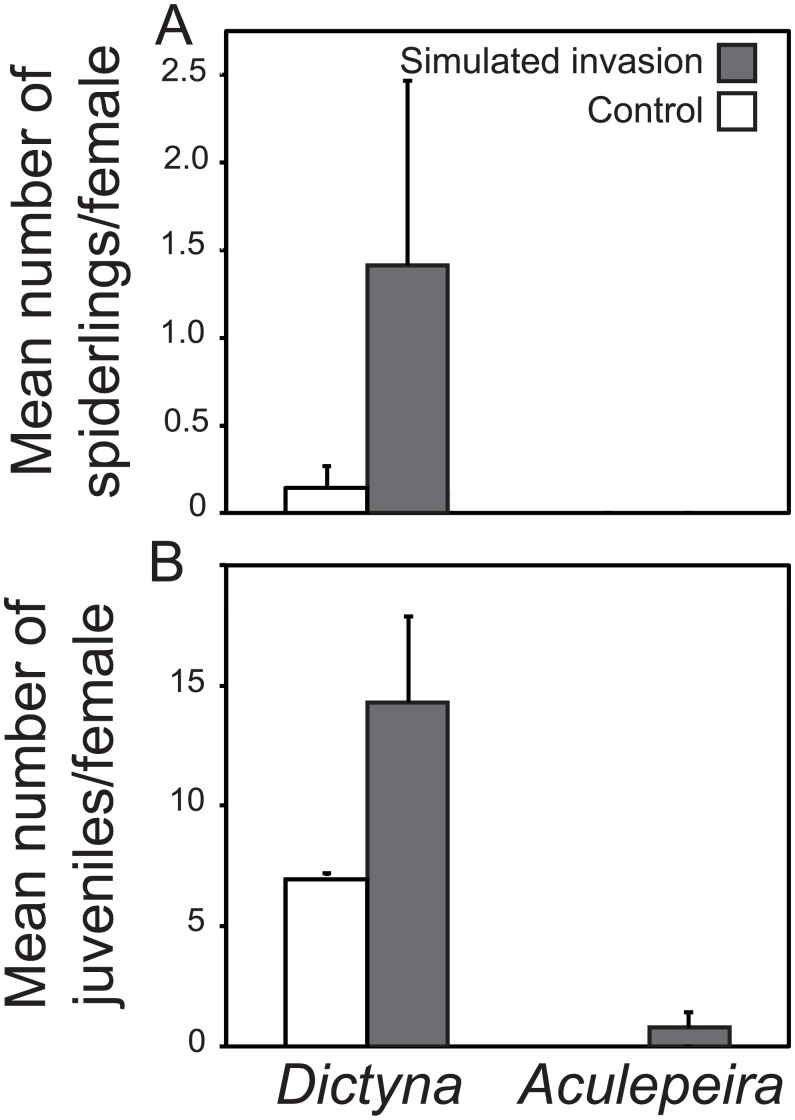
(A) Mean number of spiderlings per female (mean ± SE) in 2011 and (B) mean number of juveniles per female (mean ± SE, raw data) in 2012 for *Dictyna* and *Aculepeira*. There were more *Dictyna* juveniles present and spiderlings tended to be higher on simulated invasion plots compared to controls. We saw no differences in reproduction by treatment for *Aculepeira* (see Results).

Overwintering survival did not differ for *Dictyna* on treatment versus control plots (*F*_*1*,*2*_ = 0.015, *P* = 0.913). During sampling in July and August 2011, *Dictyna* webs on treatment plots tended to have more spiderlings compared with on control plots (Table 1, Fig 5A). Timing of sampling in 2012 missed capturing this metric for *Dictyna*, thus only 2011 data are presented. *Aculepeira* spiderlings disperse rapidly from their natal web, making quantification of this reproduction metric unfeasible. Thus, interspecific comparisons of reproduction were conducted on the mean number of juveniles per female, which was shown to be higher on treatment compared to control plots for *Dictyna*, but not for *Aculepeira* (Table 1, Fig 5B). *T*. *laboriosa* only colonized simulated invasion plots, so formal statistical analyses were not possible for web size, prey capture rates, and recruitment.

## Discussion

Exotic species invasions restructure recipient communities around the world, yet we seldom understand the mechanisms driving native species responses to invasions. Here, we use a large-scale simulated invasion experiment to show that native web spider population responses to *C*. *stoebe* invasions can be explained as a function of how key invader traits interact with species-specific traits of the natives. By introducing only dead stems of *C*. *stoebe* into native grasslands, we caused dramatic increases in native web spider populations that paralleled those documented following natural invasions [13]. This result suggests that *C*. *stoebe*’s architecture is the primary trait causing native web spider responses to invasion. We also show that the mechanism underlying differences in species-specific responses to simulated invasion derived from differences in web-building behavior, particularly as they relate to web substrate constraints in the native system. While web-spiders generally exhibited strong population responses to the simulated invasion treatment, the strongest population response in *Dictyna* was additionally associated with increased web size, higher prey captures, elevated recruitment, and greater release from substrate constraints. Examining in detail how these web-building behaviors relate to demographic and population responses of native spiders to invasion helps to elucidate key factors (i.e. web substrate quantity and quality) constraining and structuring the native web-spider community.

One of the greatest barriers to understanding biological invasions is the challenge of experimentally manipulating invasions at appropriate spatial scales. Large-scale, *in situ* experimental examinations of invasions are rare due to logistical, economical, and ethical constraints, but such studies are necessary to fully understand outcomes of perturbations like species introductions (e.g., [24]). While we overcame many of these constraints, we were still limited to three replicate sites, which limited our ability to demonstrate clear statistically significant differences in web spider populations between simulated invasion and control plots. Nonetheless, the degree of differences in population responses and consistency with observations following natural invasions [13] suggest that these findings are biologically quite significant. Moreover, our experimental treatment was conservative and basically simulated earlier stages of invasion given that the *C*. *stoebe* stem densities we used were two orders of magnitude lower than the densities *C*. *stoebe* can achieve through natural invasion [21].

The irregular web spiders, *Dictyna*, showed the strongest positive responses to simulated invasion. Final *Dictyna* mean densities were 841 spiders/0.25 ha on simulated invasion plots versus 27 spiders/0.25 ha on control plots (>30 times higher densities on treatment). The orb weaving spiders, *Aculepeira*, also showed strong positive population responses following simulated invasion. *Aculepeira* mean densities were 31 spiders/0.25 ha on simulated invasion grids versus 1 spider/0.25 ha on control grids (>23 times higher densities on treatment) and *Tetragnatha* naturally colonized only simulated invasion grids. Overall, spider population responses suggested that initial seeding densities were reasonable and that the study duration was sufficient to capture equilibrium responses to initial stages of invasion for *Aculepeira* on both treatment and control plots and for *Dictyna* on controls. On control grids, *Dictyna* densities fluctuated but remained fairly stable, ending at levels slightly higher than seeding densities. This result suggests that initial seeding densities approximated native carrying capacity for *Dictyna*. In contrast, *Aculepeira* declined rapidly on control plots to stabilize at very low densities, suggesting that initial seeding densities were likely high despite attempts to adjust for the lower natural densities of this species when seeding the plots. The addition of a non-manipulated (= no spiders added) reference plot at each site would have allowed quantitative assessment of initial seeding densities on control plots and provided stronger inference into if final spider densities had indeed reached a new equilibrium. On experimentally invaded plots, the populations of both species increased substantially, but differed in that *Aculepeira* densities appeared to stabilize by the end of the experiment while *Dictyna* were still increasing (Fig 3). This result suggests that at the termination of the experiment *Aculepeira* densities approximated their maximal response to the treatment, while *Dictyna* populations were still increasing, despite their dramatic positive population response over the first two years. It is important to note that our study ran for only two years and it is possible that competition for prey or web substrates could become important factors as one or both species continue to increase in abundance over time. For example, *Aculepeira* constructing webs on simulated invasion plots tended to be less likely to capture prey compared to *Aculepeira* on control plots. This result suggests that *Aculepeira* populations may become prey limited as *Dictyna* populations continue to increase. However, observational studies suggest that over the long term both species increase substantially in invaded compared to uninvaded habitats.

Identifying the key invader attributes restructuring the recipient community is a prerequisite to linking native species traits to their responses to invasion. Of course, key invader traits will differ as a function of the native species being considered. Invasion by *C*. *stoebe* affects many community attributes, including plant diversity and productivity, invertebrate abundance and composition, and abundance of birds and small mammals [22–24, 39–41], all of which could affect spider populations. However, by experimentally isolating *C*. *stoebe*’s plant architecture from all other aspects of its invasion, we established that changes in architecture alone are sufficient to drive increases in native web spider populations to levels that mimic those observed following *C*. *stoebe* invasions. This outcome confirms that *C*. *stoebe* affects native spider populations by serving as an invasive ecosystem engineer in this system [17]. In particular, *C*. *stoebe* invasion in these grasslands alters two important aspects of vegetation architecture that reflect important attributes of spider habitat—substrate quantity and quality. Increased substrate quantity is likely the primary driver of the dramatic increases in web-spider abundance or at least the necessary first step, given the severity of substrate constraints in this system [13, 21]. Increasing substrate availability for sessile organisms with high reproductive outputs commonly results in population increases due to release from substrate limitation (see [42]). Not surprisingly then, other invasive ecosystem engineers like zebra mussels (*Dreissena* spp.) have been shown to dramatically increase the abundance and richness of native species by reducing substrate limitation [43]. However, within our system, the higher quality (e.g., larger, more expansive, and more persistent architecture; Fig 1B) of *C*. *stoebe* substrates also allowed *Dictyna* to build 4.4 times larger webs that captured 5.0 times more prey and more than doubled their chances of reproduction, suggesting substrate quality may also feed into this process. *Aculepeira* showed no indication of change in web size or reproductive output on simulated invasion plots compared to controls, indicating that increases in their abundance, at least in simulated early stages of invasion, was due solely to increased substrate quantity releasing them from substrate limitation. Given that *Aculepeira* may have experienced reductions in prey captures on simulated invasion plots, it is possible that resource competition could limit their populations in later stages of invasion.

An important question is, why do these spider species respond so differently to invasion, and what can such invasions teach us about native community structuring? The substantial differences in the strength of the responses of *Dictyna* compared to *Aculepeira* following release from substrate limitation appeared to be driven by differences in their web-building behaviors relative to substrate constraints present in their native pre-invasion system. Because *Dictyna* construct their webs entirely within individual plants and most native plant species in our system provide diminutive substrates for this species, web-building in these irregular web spiders is severely constrained and every new *C*. *stoebe* stem offers a functionally viable web substrate that also allows it to plastically increase its web size and improve its fitness through higher prey captures. This phenomenon may extend to other spiders in this system that use individual plant substrates as well. However, orb weavers like *Aculepeira*, *Tetragnatha*, and others that use multiple plants to suspend their webs are unconstrained by individual plant characteristics, and thus are much less constrained by the quality of native plant architectures.

These findings shed light on the factors determining native spider community structuring in this system. Although spiders can be food- [15, 18, 44–47] or substrate-limited [48–50], in this system substrate limitation appears to be severe and may be the primary factor limiting native web spider diversity in these species-poor grasslands. Native spiders appear to produce more offspring than can find suitable substrates for establishment. Hence, increases in substrate quantity can have very powerful and immediate effects on the spider community. However, our results also show that species- or guild-specific web construction traits relative to substrate attributes have very important ramifications for how spider communities are ultimately structured. For the irregular web spiders like *Dictyna* and other species exhibiting plasticity in web construction, changes in substrate quality could also increase reproduction by releasing spiders from food limitation. Orb weavers in contrast are commonly more fixed in their web construction behaviors [51–53] and while orb weavers have been shown to be flexible to spatial constraints [54] they may be less capable of exploiting this type of change in vegetation architecture.

Biological invasions disrupt and reorganize communities around the world, yet we seldom understand the community-level ramifications of such invasions. This is largely because traditional approaches in invasion biology have relied heavily on observational studies. Our study demonstrates how simulated *in situ* invasions may be used to identify and understand the mechanisms restructuring native communities, while also demonstrating the inherent challenges of executing such experiments. Furthermore, our work suggests that linking functional traits of native species to invader traits (or invader-driven changes in the recipient community) can improve understandings of community reassembly following invasion. Retrospective studies like ours applied to other invaders may help to identify the mechanisms underlying invasion outcomes. Moreover, applying retrospective understandings and general niche theory to new systems may prove effective for predicting invasion outcomes. For example, exotic forb invasions into grasslands in central Argentina appear to increase the complexity of plant architecture and local densities of native web spiders in a manner very similar to what we have shown here (D.E. Pearson *pers*. *obs*.). Mechanistic understandings like those we have developed here could be applied to this system and others to test predictions for native web spider responses as well as the responses of their prey.

## Supporting Information

S1 Appendix Code for analyses run in R version 3.2.2. (DOCX)

S2 Appendix Code for analyses run in SAS version 9.2. (DOCX)

S1 Table Mean abundance (± SE) of web building spiders on simulated invasion treatment and control plots over the two-year study (2011–2012). (DOCX)

S2 Table Mean invertebrate prey abundance (± SE) from sweep netting on simulated invasion treatment and control plots at three sites (HL = Harper’s Lake, BCGR = Blackfoot Clearwater Game Range, BR = Bandy Ranch) in July 2012. (DOCX)
