# Supplementary material for: Linking Native and Invader Traits Explains Native Spider Population Responses to Plant Invasion
Source: PLoS One. 2016 Apr 15;11(4):e0153661. doi: 10.1371/journal.pone.0153661 (PMC4833385; doi:10.1371/journal.pone.0153661)
Supplement: S1 Appendix — (DOCX) [file pone.0153661.s001.docx]

**S1 Appendix. Code for analyses run in R version 3.2.2.**

**1A)** **The effect of simulated invasion treatment on spider abundance was analyzed using PERMANOVA procedures in R with treatment as a fixed factor.**

# station = indicates site (HL, BR, BCGR) and simulated invasion (T) versus control (C)

# dictyna = *Dictyna* change in abundance (August 2012 abundance – June 2011 abundance)

# aculepeira = *Aculepeira* change in abundance

# tetragnatha = *Tetragnatha* change in abundance

# trt = simulated invasion treatment or control

# create data frame

dat = data.frame("station"=c("HL-T","HL-C","BR-T","BR-C","BCGR-T","BCGR-C"),

"dictyna"=c(401,-5,921,5,1140,22),

"aculepiera"=c(46,-8,0,-8,17,-10),

"tetragnatha"=c(2,0,0,0,0,0),

"trt"=c("T","C","T","C","T","C"))

dat.sub = dat[,2:4]

library(vegan)

# run overall PERMANOVA analysis

out.adonis = adonis(dat.sub ~ dat$trt,method="euclidean")

out.adonis

# species specific PERMANOVA analyses

out.adonis.dictyna = adonis(dat.sub$dictyna ~ dat$trt,method="euclidean")

out.adonis.dictyna

out.adonis.aculepiera = adonis(dat.sub$aculepiera ~ dat$trt,method="euclidean")

out.adonis.aculepiera

out.adonis.tetragnatha = adonis(dat.sub$tetragnatha ~ dat$trt,method="euclidean")

out.adonis.tetragnatha

####################

**1B) The effect of simulated invasion treatment on overwintering survival in *Dictyna* was analyzed using a linear mixed effects model (LMM) in R (package nlme) with treatment as a fixed effect and site as a random blocking factor.**

#dict.ow = *Dictyna* overwintering survival (June 2012 abundance – August 2011 abundance)

#site = three sites where study was conducted [Harper’s Lake (HL), Blackfoot Clearwater Game Range (BCGR), and Bandy Ranch (BR)]

#fits a linear mixed effects model (LMM) looking at the effect of plot on *Dictyna* overwintering

survival with site as a random blocking factor

model.dict.overwinter <- lme(dict.ow ~ plot, random = ~ 1|site)

#checking normality assumption

#q-q plot

qqnorm(resid(model.dict.overwinter), main = "Normal Q-Q Plot Dictyna Overwinter", xlab="Theoretical Quantiles", ylab="Sample Quantiles")

#residual plot

plot(dict.overwinter$abundance, resid(model.dict.overwinter), ylab="Residuals", xlab="Abundance", main="Dictyna Overwintering Abundance")

abline(0,0)

####################

**1C) We tested for differences in average web area across sampling periods (June, July, and August) in 2011 using a LMM (package nlme) in R with plot and sampling period as fixed effects and site as a random blocking factor.**

#spider.spp = spider species (DICT *= Dictyna*, ACUL = *Aculepeira*)

#period = sampling period (June, July, August)

#log.web = log transformed web size

#*Dictyna* 2011

#subset the data to only look at *Dictyna*

dict.web.2011 <- subset(web.2011, spider.spp == "DICT", select = c(period,plot,site,log.web))

#fits a LMM looking at the effect of plot and sampling period on *Dictyna* log web size with site

as a random blocking factor

model.dict.web <-lme(log.web ~ plot + period, random=~1|site,data = dict.web.2011)

#checking normality assumption with q-q plot

qqnorm(resid(model.dict.web),main = "Normal Q-Q Plot Dictyna Webs Over Sampling Periods", xlab="Theoretical Quantiles",ylab="Sample Quantiles")

#*Aculepeira* 2011

#subset the data to only look at *Aculepeira*

acul.web.2011 <- subset(web.2011, spider.spp == "ACUL", select = c(period,plot,site,log.web))

#fits a LMM looking at the effect of plot and sampling period on *Aculepeira* log web size with

site as a random blocking factor

model.acul.web <-lme(log.web ~ plot + period, random=~1|site,data = acul.web.2011)

#checking normality assumption with q-q plot

qqnorm(resid(model.acul.web),main = "Normal Q-Q Plot Aculepeira Webs Over Sampling Periods", xlab="Theoretical Quantiles",ylab="Sample Quantiles")

####################

**1D) The effect of simulated invasion treatment on mean number of juveniles per female** $\boldsymbol{(=}\frac{\boldsymbol{\# juveniles September 2012}}{\boldsymbol{\# reproducing females June 2012}}\boldsymbol{)}$ **was analyzed using a LMM in R (package nlme) with treatment as a fixed effect and site as a random blocking factor.**

#dict.juv.abundance = mean number of juveniles per *Dictyna* female $(=\frac{\# juveniles September 2012}{\# reproducing females June 2012})$

#acul.juv.abundance = mean number of juveniles per *Aculepeira* female $(=\frac{\# juveniles September 2012}{\# reproducing females June 2012})$

#*Dictyna*

#fits a LMM looking at the effect of plot on the mean number of juveniles per female *Dictyna*

with site as a random blocking factor

model.dict.juv <- lme(dict.juv.abundance ~ plot, random = ~ 1 |site)

#checking normality assumption

#q-q plot

qqnorm(resid(model.dict.juv),main = "Normal Q-Q Plot Dictyna Recruitment", xlab="Theoretical Quantiles",ylab="Sample Quantiles")

#residual plot

plot(dict.juv.abundance, resid(model.dict.juv), ylab="Residuals", xlab="Abundance", main="Dictyna Recruitment")

abline(0,0)

#*Aculepeira*

#fits a LMM looking at the effect of plot on the mean number of juveniles per female *Aculepeira*

with site as a random blocking factor

model.acul.juv <- lme(acul.juv.abundance ~ plot, random = ~ 1|site)

#checking normality assumption

#q-q plot

qqnorm(resid(model.acul.juv),main = "Normal Q-Q Plot Aculepeira Recruitment", xlab="Theoretical Quantiles",ylab="Sample Quantiles")

#residual plot

plot(acul.juv.abundance, resid(model.dict.juv), ylab="Residuals", xlab="Abundance", main="Aculepeira Recruitment")

abline(0,0)

**1E) The effect of simulated invasion treatment on available prey was analyzed using a LMM in R (package nlme) with treatment as a fixed effect and site as a random blocking factor.**

#mean.prey.abundance = mean number of prey per 10 sweep net samples

#fits a LMM looking at the effect of plot on the mean number of invertebrate prey with site as a

random blocking factor

model.prey <- lme(mean.prey.abundance ~ plot, random = ~1|site)

#checking normality assumption

#q-q plot

qqnorm(resid(model.prey),main = "Normal Q-Q Plot Prey Abundance", xlab="Theoretical Quantiles",ylab="Sample Quantiles")

#residual plot

plot(mean.prey$mean.prey.abundance, resid(fm.prey.1), ylab="Residuals", xlab="Mean Prey Abundance", main="Prey Abundance")

abline(0,0)
