# Supplementary material for: Linking Native and Invader Traits Explains Native Spider Population Responses to Plant Invasion
Source: PLoS One. 2016 Apr 15;11(4):e0153661. doi: 10.1371/journal.pone.0153661 (PMC4833385; doi:10.1371/journal.pone.0153661)
Supplement: S2 Appendix — (DOCX) [file pone.0153661.s002.docx]

**S2 Appendix. Code for analyses run in SAS version 9.2.**

**2A) The effect of simulated invasion treatment on average web area was analyzed with a LMM using PROC GLIMMIX in SAS, with treatment and year as fixed effects, site and year as random factors, and a lognormal distribution.**

TITLE 'DICT WEB AREA';

**PROC** **GLIMMIX** DATA=SPIDER;

CLASS TREAT SITE YEAR;

WHERE SPECIES = 'DICT' AND SEX NE 'M';

MODEL AREA = TREAT YEAR TREAT*YEAR /DIST=LOGNORMAL;

RANDOM SITE*TREAT SITE*TREAT*YEAR;

**RUN**;

TITLE 'ACULAPEIRA WEB AREA'; *NS;

**PROC** **GLIMMIX** DATA=SPIDER;

CLASS TREAT SITE YEAR;

WHERE SPECIES = 'ACUL' AND SEX NE 'M';

MODEL AREA = TREAT YEAR TREAT*YEAR/DIST=LOGNORMAL;

RANDOM SITE*TREAT SITE*TREAT*YEAR;

NLOPTIONS MAXIT=**200** TECH=NRRIDG; ** options to help with convergence;

**RUN**;

**2B)** **The effect of simulated invasion treatment on number of prey captured per web** **was analyzed with a LMM using PROC GLIMMIX in SAS, with treatment and year as fixed effects, and site and year as random factors. To meet assumptions of normality and equal variance, number of prey was analyzed using a negative binomial distribution.**

TITLE1 'DICT NUMBER OF PREY CAPS';

**PROC** **GLIMMIX** DATA=SPIDER;

CLASS TREAT SITE YEAR;

WHERE SPECIES = 'DICT' AND SEX NE 'M' ;

MODEL NUM_PREY = TREAT YEAR TREAT*YEAR/ DIST=NEGBIN;

RANDOM SITE*TREAT SITE*TREAT*YEAR;

NLOPTIONS MAXIT=**200** TECH=NRRIDG; ** options to help with convergence;

LSMEANS TREAT TREAT*YEAR/ILINK;

**RUN**;

TITLE1 'ACUL NUMBER OF PREY CAPS';

**PROC** **GLIMMIX** DATA=SPIDER;

CLASS TREAT SITE YEAR;

WHERE SPECIES = 'ACUL' AND SEX NE 'M' ;

MODEL NUM_PREY = TREAT YEAR TREAT*YEAR/ DIST=NEGBIN;

RANDOM SITE*TREAT SITE*TREAT*YEAR;

NLOPTIONS MAXIT=**200** TECH=NRRIDG; ** options to help with convergence;

LSMEANS TREAT TREAT*YEAR/ ILINK;

**RUN**;

**2C)** **The effect of simulated invasion treatment on the probability of capturing prey** **was analyzed with a LMM using PROC GLIMMIX in SAS with treatment and year as fixed effects, and subplot within site and year as random factors. The probability of capturing prey was analyzed using a binomial distribution.**

* CREATE VARIABLE TO EVALUATE WHETHER SPIDERS CAUGHT PREY OR NOT;

**data** spider;

set spider;

PREYCAP = **0**;

if NUM_PREY >**0** then PREYCAP = **1**;

**run**;

TITLE1 'DICT PREY CAPS YES/NO';

**PROC** **GLIMMIX** DATA=SPIDER;

CLASS TREAT SITE YEAR;

WHERE SPECIES = 'DICT' AND SEX NE 'M' ;

MODEL PREYCAP = TREAT YEAR TREAT*YEAR/ DIST=BIN;

RANDOM SITE*TREAT SITE*TREAT*YEAR;

NLOPTIONS MAXIT=**200** TECH=NRRIDG; ** options to help with convergence;

**RUN**;

TITLE1 'ACUL PREY CAPS YES/NO';

**PROC** **GLIMMIX** DATA=SPIDER;

CLASS TREAT SITE YEAR;

WHERE SPECIES = 'ACUL' AND SEX NE 'M' ;

MODEL PREYCAP = TREAT YEAR TREAT*YEAR/ DIST=BIN;

RANDOM SITE*TREAT SITE*TREAT*YEAR;

NLOPTIONS MAXIT=**200** TECH=NRRIDG; ** options to help with convergence;

**RUN**;

**2D) The effect of simulated invasion treatment on the probability of capturing large prey (≥ 3 mm for *Dictyna*, ≥ 4 mm for *Aculepeira*)** **was analyzed with a LMM using PROC GLIMMIX in SAS with treatment and year as fixed effects, and subplot within site and year as random factors. The probability of capturing prey was analyzed using a binomial distribution.**

* CREATE VARIABLES TO EVALUATE WHETHER SPIDERS CAUGHT LARGE PREY OR NOT;

**data** spider;

set spider;

PREY3 = **0**;

if ave_prey_size >**3** then PREY3 = **1**;

**run**;

**data** spider;

set spider;

PREY4 = **0**;

if prey_4mm >**0** then PREY4 = **1**;

**run**;

TITLE2 'DICT BIGPREY CAPS';

**PROC** **GLIMMIX DATA=SPIDER**;

CLASS TREAT SITE YEAR;

WHERE SPECIES = 'DICT' AND SEX NE 'M' ;

MODEL prey3 = TREAT YEAR TREAT*YEAR/ DIST=bin;

RANDOM SITE*TREAT SITE*TREAT*YEAR;

NLOPTIONS MAXIT=**200** TECH=NRRIDG; ** options to help with convergence;

LSMEANS TREAT TREAT*YEAR/ILINK;

**RUN**;

TITLE2 'ACUL BIGPREY CAPS';

**PROC** **GLIMMIX DATA=SPIDER**;

CLASS TREAT SITE YEAR;

WHERE SPECIES = 'ACUL' AND SEX NE 'M' ;

MODEL prey4 = TREAT YEAR TREAT*YEAR/ DIST=bin;

RANDOM SITE*TREAT SITE*TREAT*YEAR;

NLOPTIONS MAXIT=**200** TECH=NRRIDG; ** options to help with convergence;

LSMEANS TREAT TREAT*YEAR/ILINK;

**RUN**;

**2E) The effect of simulated invasion treatment on the number of spiderlings per female *Dictyna* in 2011** **was analyzed using a LMM using PROC GLIMMIX in SAS, with treatment as a fixed effect and plot within site as a random factor using the negative binomial distribution.**

TITLE1 'DICT SPIDERLINGS';

**PROC** **GLIMMIX DATA=SPIDER**;

CLASS TREAT SITE YEAR;

WHERE SPECIES = 'DICT' AND SEX NE 'M' AND YEAR = **2011**;

MODEL num_spiderlings = TREAT / DIST=negbin;

RANDOM SITE*TREAT ;

NLOPTIONS MAXIT=**200** TECH=NRRIDG; ** options to help with convergence;

**RUN**;
