# Supplementary material for: Linking Native and Invader Traits Explains Native Spider Population Responses to Plant Invasion
Source: PLoS One. 2016 Apr 15;11(4):e0153661. doi: 10.1371/journal.pone.0153661 (PMC4833385; doi:10.1371/journal.pone.0153661)
Supplement: S1 Table — (DOCX) [file pone.0153661.s003.docx]

**S1 Table. Mean abundance (± SE) of web building spiders on simulated invasion treatment and control plots over the two-year study (2011-2012).**

|  | | ***Dictyna*** | | ***Aculepeira*** | | ***Tetragnatha*** | |
| --- | --- | --- | --- | --- | --- | --- | --- |
|  |  | Treatment | Control | Treatment | Control | Treatment | Control |
| 2011 | Seeding | 20 | 20 | 10 | 10 | 0 | 0 |
|  | June | 32.667 ± 12.252 | 26.333 ± 7.311 | 19.333 ± 6.119 | 8.0 ± 6.557 | 7.333 ± 3.528 | 0.333 ± 0.333 |
|  | July | 18.333 ± 5.487 | 11.0 ± 6.110 | 6.333 ± 1.764 | 2.667 ± 1.453 | 2.0 ± 0.577 | 0 |
|  | August | 66.333 ± 25.543 | 11.333 ± 6.566 | 6.000 ± 2.082 | 2.0 ± 1.155 | 2.0 ± 1.0 | 0 |
| 2012 | June | 64.333 ± 21.302 | 5.0 ± 1.528 | 37.0 ± 10.017 | 2.0 ± 0.577 | 2.667 ± 1.764 | 0 |
|  | July | 438.667 ± 107.372 | 6.667 ± 2.603 | 7.333 ± 2.963 | 0.667 ± 0.333 | 0.333 ± 0.333 | 0 |
|  | August | 840.667 ± 212.289 | 27.333 ± 7.881 | 31.0 ± 13.429 | 1.333 ± 0.667 | 0.667 ± 0.667 | 0 |
