# Supplementary material for: Linking Native and Invader Traits Explains Native Spider Population Responses to Plant Invasion
Source: PLoS One. 2016 Apr 15;11(4):e0153661. doi: 10.1371/journal.pone.0153661 (PMC4833385; doi:10.1371/journal.pone.0153661)
Supplement: S2 Table — (DOCX) [file pone.0153661.s004.docx]

**S2 Table. Mean invertebrate prey abundance (± SE) from sweep netting on simulated invasion treatment and control plots at three sites (HL = Harper’s Lake, BCGR = Blackfoot Clearwater Game Range, BR = Bandy Ranch) in July 2012.**

|  | **Treatment** | **Control** |
| --- | --- | --- |
| HL | 4.5 ± 0.778 | 5.0 ± 0.919 |
| BCGR | 5.4 ± 1.096 | 7.6 ± 1.310 |
| BR | 14.8 ± 5.072 | 15.6 ± 3.670 |
